# Supplementary material for: Clinical Introduction of Stem Cell Sparing Radiotherapy to Reduce the Risk of Xerostomia in Patients with Head and Neck Cancer
Source: Cancers (Basel). 2024 Dec 23;16(24):4283. doi: 10.3390/cancers16244283 (PMC11674908; doi:10.3390/cancers16244283)
Supplement: Supplementary file 1 [file cancers-16-04283-s001.zip › cancers-3305862-supplementary.pdf]

Supplementary Materials: Clinical Introduction of Stem Cell Sparing Radiotherapy to Reduce the Risk of Xerostomia in Patients with Head and Neck Cancer

Maria I. van Rijn-Dekker, Arjen van der Schaaf, Sanne W. Nienhuis, Antoinette S. Arents-Huls, Rachel G. Ger, Olga Hamming-Vrieze, Frank J.P. Hoebers, Mischa de Ridder, Sabrina Vigorito, Ellen M. Zwijnenburg, Johannes A. Langendijk, Peter van Luijk and Roel J.H.M. Steenbakkers

Table S1. Clinical goals for target coverage.

	Photon therapy	Proton therapy
**High dose target**		
At **least** 98.00% volume at 66.50 Gy (RBE) dose	+ (PTV_{70})	+ (CTV_{70})
At **least** 98.00% volume at 65.80 Gy (RBE) dose	-	+ (CTV_{70})
At **least** 69.50 Gy (RBE) average dose	+ (PTV_{70})	+ (CTV_{70})
At **most** 2.00% volume at 74.90 Gy (RBE) dose	+ (PTV_{70})	+ (PTV_{70})
At **most** 70.50 (RBE) Gy average dose	+ (PTV_{70})	+ (CTV_{70})
**Elective target**		
At **least** 98.00% volume at 51.54 Gy (RBE) dose	+ ($PTV_{54.24}$)	+ ($CTV_{54.25}$)
At **least** 98.00% volume at 51.00 Gy (RBE) dose	-	+ ($CTV_{54.25}$)

Abbreviations: CTV = clinical target volume; Gy = Gray; PTV = planning target volume; RBE = relative biological effectiveness.

Table S2. Organs at risk delineated for study evaluation.

**Parotid gland structures**
➢ Whole parotid glands
➢ SCR regions
➢ Non-SCR regions (i.e., remaining parotid gland tissue [parotid gland minus SCR regions])
**Other salivary OARs**
➢ Submandibular glands
➢ Oral cavity
**Swallowing OARs**
➢ Superior, middle, and inferior PCM
➢ Supraglottic area
**Other OARs**
➢ Brain
➢ Brainstem

All OARs were delineated according to published guidelines.[1–3] *Abbreviations:* OAR = organ at risk; PCM = pharyngeal constrictor muscle; SCR = stem cell rich.

Table S3. NTCP models to estimate clinical impact.

	Toxicity	Questionnaire	Published by
Expected benefit from SCS-RT	Patient-reported daytime xerostomia	GRIX	van Rijn-Dekker et al.
	Patient-reported eating-related xerostomia	GRIX	van Rijn-Dekker et al.
	Physician-reported grade ≥2 xerostomia	CTCAE	van Rijn-Dekker et al.
Expected consequences of dose shifts to other OARs_	Physician-reported grade ≥2 dysphagia	CTCAE	Van den Bosch et al.
	Patient-reported aspiration	EORTC QLQ-H&N35	Van den Bosch et al.
	Patient-reported xerostomia	EORTC QLQ-H&N35	Van den Bosch et al.
	Patient-reported sticky saliva	EORTC QLQ-H&N35	Van den Bosch et al.
	Patient-reported taste loss	EORTC QLQ-H&N35	Van den Bosch et al.
	Patient-reported speech problems	EORTC QLQ-H&N35	Van den Bosch et al.
	Patient-reported oral pain	EORTC QLQ-H&N35	Van den Bosch et al.
	Patient-reported nausea & vomiting	EORTC QLQ-C30	Van den Bosch et al.
	Patient-reported fatigue	EORTC QLQ-C30	Van den Bosch et al.

Van Rijn-Dekker et al. developed NTCP models for xerostomia outcomes at 6, 12, and 24 months after RT[4], while Van den Bosch et al. developed NTCP models for several toxicities at 6, 12, 18, and 24 months after RT[5]. To allow comparison of the overall estimated clinical impact, the NTCPs for 6, 12, and 24 months after RT were averaged. *Abbreviations:* CTCAE = Common Terminology Criteria for Adverse Events; EORTC = European Organization for Research and Treatment for Cancer; GRIX = Groningen Radiotherapy-Induced Xerostomia; NTCP = normal tissue complication probability; RT = radiotherapy; SCR = stem cell rich; QLQ-C30/H&N35 = Quality of Life Questionnaire Core/Head & Neck.

Table S4. Details about used optimization strategies by different centers.

	Treatment planning system	RT technique	Optimization strategy	Considered OARs (including prioritization)	Changes made in SCS-RT plan
Center 1	ST-RT: RayStation Clinical 11b<br>SCS-RT: RayStation Development v12.0.100.0	VMAT with 2 full arcs	Stepwise optimization of all objectives. Objectives for OARs and out-structures of OARs included dose fall off (i.e., reduce dose within a defined range from target) and maximum EUD.	(1) Maximum dose for brainstem and myelum<br>(2) PCMs, oral cavity, cricopharyngeal muscle<br>(3) Parotid glands, submandibular glands<br>(4) Supraglottic area, base of tongue, thyroid	Addition of SCR regions between priority (2) and (3).<br>Addition of non-SCR regions in priority (3) to have more control during optimization.
Center 2	Eclipse Treatment Planning System V15.6	VMAT with 2 arcs	Stepwise optimization of all objectives, which was stopped when mean parotid gland dose was 26-28 Gy or if target coverage was compromised.	(1) Spinal cord, parotid glands<br>(2) Brain(stem), glottic area, mandible, oral cavity, PCM, submandibular glands (if outside PTV)<br>(3) Help structures to reduce low dose anterior and posterior	Addition of SCR regions with cropped structure (minus PTV with 3 mm margin) in priority (1). Optimization was stopped if target coverage was compromised.
Center 3	Raystation Clinical 2023B	VMAT with 2 arcs	Stepwise optimization of all objectives with DVH points, which is stopped when the plan is considered clinically acceptable and no additional dose reduction in OARs is considered feasible.	(1) OARs in the midline (e.g., PCMs, oral cavity, (supra)glottic area), brainstem, spinal cord<br>(2) Parotid glands<br>(3) Submandibular glands	Addition of SCR regions (and if necessary non-SCR regions) with same priority as parotid glands. Additional margins were created to avoid competition between dose reduction in the parotid glands and SCR regions.
Center 4	Eclipse Treatment Planning System v16.01.10	VMAT with 2 arcs	*Step 1*: a plan was optimized to cover only the PTV.<br>*Step 2*: OARs were added, in which D_{max} of seriel OARs and D_{mean} of OARs_exPTV was stepwise reduced.	All delineated OARs (in practice, all head-and-neck OARs). No prioritization needed (all ALARA).	Replacement of parotid glands by SCR and non-SCR regions.
Center 5	Pinnacle v16.2.0	VMAT with 2 arcs	*Automatic planning*: all objectives were simultaneously optimized with clinical goals and dose thresholds for OARs.<br>*Manual adjustments*: to improve target coverage and increase OAR sparing, if considered feasible.	(1) Spinal cord, brainstem, parotid glands<br>(2) Larynx, oral cavity, submandibular glands, PCMs<br>(3) Help structures for conformal dose and dose reduction in neck	Addition of SCR regions (before starting automatic optimization) with dose threshold in line with thresholds for parotid glands and other OARs.

	Treatment planning system	RT technique	Optimization strategy	Considered OARs (including prioritization)	Changes made in SCS-RT plan
Center 6	Pinnacle v16.0.2	VMAT with 2 full arcs	*Semi-automatic planning*: D_{mean} of OARs_exPTV was stepwise reduced.<br>*Manual adjustments*: additional objectives when needed.	(1) Parotid glands (including an additional out-structure to gain more dose reduction)<br>(2) Other head-and-neck OARs	Replacement of additional out-structure of parotid glands by SCR regions.<br>Replacement of parotid glands by non-SCR regions.
Center 7	RayStation Clinical v12A	VMAT with 2 or 3 arcs	*Basic plan*: autoplanning with RayStation deep learning optimization.<br>*Manual adjustments*: for further optimization, including more OARs.	*In basic plan*: all delineated OARs, of which the swallowing muscles and salivary glands are prioritized.<br>*Manual adjustments*: minimize the total NTCP for xerostomia and dysphagia.	Replacement of parotid glands by SCR and non-SCR regions.
Center 8	Monaco v6.00.01	VMAT	Biological cost functions, in which primarily D_{mean} of OARs was reduced, using Monte Carlo dose calculation.	(1) Salivary glands<br>(2) Oral cavity<br>(3) Brainstem, cricopharyngeal muscle, mandible, esophagus, PCMs, spinal cord, (supra)glottic area, thyroid, trachea	Addition of SCR regions in priority (1).

Abbreviations: ALARA = as low as reasonably achievable; D_{mean} = mean dose; EUD = equivalent uniform dose; non-SCR regions = remaining parotid gland tissue (i.e., parotid gland minus SCR region); OAR = organ at risk; OARs_exPTV = OAR minus PTV; out-structures = OAR minus PTV with 5 mm margin[6]; PTV = planning target volume; PCM = pharyngeal constrictor muscle; SCR = stem cell rich; VMAT = volumetric arc modulated therapy.

Table S5. Different SCS-RT strategies from 3 RTTs (step 1): comparison of mean dose (in Gy) between SCS-RT and ST-RT.

	PHOTONS (n=5)		PROTONS (n=5)		P value
	RTT1 median [range]	RTT2 median [range]	RTT1 median [range]	RTT3 median [range]	
**Parotid gland**					
IL SCR region	-4.4 [-6.3;-0.3]	-2.0 [-3.2;-1.7]	-2.2 [-6.3;-0.7]	-1.1 [-6.4;-0.1]	0.72
CL SCR region	-4.1 [-6.9;-1.2]	-1.7 [-5.0;3.1]	-3.6 [-5.8;-0.3]	-2.2 [-5.3;-0.5]	0.36
IL parotid gland	-3.3 [-5.5;0.1]	-0.2 [-1.3;2.1]	-2.3 [-5.0;0.1]	-0.7 [-5.7;0.0]	0.56
CL parotid gland	-4.1 [-5.7;-1.1]	1.3 [-2.7;3.3]	-1.8 [-4.2;0.0]	-1.1 [-4.3;-0.2]	0.15
IL non-SCR region	-3.2 [-5.2;0.3]	0.2 [-1.5;3.5]	-2.3 [-4.5;0.2]	-0.5 [-5.6;0.0]	0.05
CL non-SCR region	-4.2 [-5.0;-1.0]	1.9 [-1.9;3.5]	-1.1 [-3.7;0.2]	-0.7 [-4.0;0.0]	0.05
**Other organs at risk**					
IL submandibular gland	-0.1 [-2.6;1.8]	-0.3 [-3.7;0.6]	-0.1 [-0.2;1.9]	-0.8 [-1.9;0.1]	0.47
CL submandibular gland	-0.3 [-1.7;4.1]	-0.4 [-2.9;1.1]	0.0 [-0.2;1.3]	-0.4 [-4.5;0.5]	0.22
Oral cavity	0.4 [-0.5;4.5]	1.9 [-1.5;7.1]	1.2 [-0.0;2.0]	-0.2 [-1.5;0.2]	0.32
Superior PCM	0.5 [-0.5;1.3]	-0.2 [-0.5;0.4]	0.1 [-0.5;1.1]	-0.7 [-2.1;0.5]	0.18
Middle PCM	0.6 [-3.2;1.4]	2.6 [0.3;4.9]	0.2 [-1.2;2.5]	-0.4 [-5.0;1.0]	0.11
Inferior PCM	-0.1 [-0.8;1.5]	4.1 [0.6;5.5]	-0.0 [-1.0;1.1]	-0.8 [-3.0;0.5]	0.03*
Supraglottic area	-1.8 [-4.2;1.8]	-1.9 [-3.0;7.0]	0.4 [-2.4;3.7]	-2.0 [-7.7;-0.1]	0.18
Brain	0.3 [0.0;0.6]	-0.1 [-0.2;0.0]	0.6 [-0.1;2.6]	0.0 [-1.9;0.5]	0.04*
Brainstem	0.5 [0.2;3.6]	1.6 [-1.7;4.4]	0.4 [-1.9;3.0]	-0.2 [-1.3;0.2]	0.07

This table depicts the mean dose differences (in Gy) between SCS-RT and ST-RT for photon and proton therapy (i.e., mean dose in ST-RT plan minus mean dose in SCS-RT plan). The ST-RT plans were the clinical plans. *Abbreviations:* CL = contralateral (i.e., receiving lowest dose); Gy = Gray; IL = ipsilateral (i.e., receiving highest dose); non-SCR = remaining parotid gland tissue (i.e., parotid gland minus SCR region); PCM = pharyngeal constrictor muscle; RT = radiotherapy; RTT = radiotherapy technologist; SCR = stem cell rich; SCS = stem cell sparing; ST = standard. * Significant according to Friedman test (significance level of $p<0.05$).

Table S6. Dose shifts due to SCS-RT (step 2 and 4): comparison of mean dose (in Gy) with photons and protons.

	PHOTONS (n=30)				PROTONS (n=15)			
	ST-RT median [range]	SCS-RT median [range]	Dose difference median [range]	P value	ST-RT median [range]	SCS-RT median [range]	Dose difference median [range]	P value
**Parotid gland**								
IL SCR region	23.2 [11.6;64.9]	17.2 [8.3;63.5]	-4.1 [-13.0;1.3]	<0.001*	18.2 [7.3;63.0]	16.3 [5.8;60.8]	-2.2 [-6.3;-0.7]	<0.001*
CL SCR region	15.3 [8.0;32.7]	11.8 [7.0;26.6]	-3.5 [-19.1;1.0]	<0.001*	10.5 [3.5;22.7]	7.1 [2.4;19.1]	-1.9 [-5.8;0.9]	0.002*
IL parotid gland	25.8 [13.6;57.2]	25.1 [10.9;57.3]	-2.8 [-9.3;0.8]	<0.001*	21.3 [8.9;55.6]	18.9 [8.1;53.4]	-1.0 [-5.0;0.1]	<0.001*
CL parotid gland	20.2 [10.0;34.9]	17.2 [9.4;26.8]	-1.9 [-12.3;0.8]	<0.001*	14.8 [5.5;21.1]	13.7 [5.3;20.4]	-1.1 [-4.2;1.9]	0.04*
IL non-SCR region	28.6 [14.5;54.5]	26.3 [12.1;54.8]	-2.7 [-8.6;0.6]	<0.001*	20.8 [9.3;52.5]	19.9 [8.6;50.1]	-0.7 [-4.5;0.5]	0.002*
CL non-SCR region	21.7 [11.0;35.4]	19.0 [10.4;28.9]	-1.5 [-10.8;0.9]	<0.001*	14.8 [6.0;22.6]	15.3 [6.1;21.9]	-0.5 [-3.7;2.3]	0.09
**Other organs at risk**								
IL submandibular gland	54.7 [36.2;68.9]	54.7 [33.2;69.3]	-0.6 [-7.9;2.2]	0.02*	52.9 [30.1;67.5]	53.6 [29.8;67.9]	0.4 [-2.2;5.7]	0.09
CL submandibular gland	40.7 [0.0;66.1]	37.7 [0.0;65.2]	-1.3 [-6.6;4.1]	0.003*	35.0 [0.0;52.1]	37.1 [0.0;52.2]	0.4 [-4.2;8.6]	0.11
Oral cavity	29.1 [10.2;68.2]	28.7 [10.6;67.4]	-0.9 [-6.5;5.6]	0.14	22.6 [7.5;42.4]	23.0 [7.3;43.1]	0.2 [-0.6;2.0]	0.01*
Superior PCM	50.4 [20.8;69.4]	48.9 [18.7;70.3]	0.3 [-6.0;1.3]	0.48	57.8 [8.8;68.6]	57.4 [9.8;68.9]	0.1 [-0.8;4.5]	0.61
Middle PCM	46.3 [24.8;70.3]	54.4 [21.9;70.8]	-0.3 [-4.2;2.7]	0.10	40.0 [6.7;69.9]	41.1 [7.8;70.0]	0.2 [-4.5;2.5]	0.39
Inferior PCM	30.7 [19.7;69.4]	28.7 [20.8;70.1]	-0.1 [-5.2;2.6]	0.23	16.4 [9.4;70.0]	18.4 [8.4;70.2]	0.0 [-2.8;3.8]	0.82
Supraglottic area	41.4 [14.9;70.3]	39.0 [16.2;70.2]	-0.9 [-9.6;3.3]	0.001*	20.1 [2.9;68.9]	21.7 [3.5;67.2]	-0.1 [-5.1;3.7]	0.82
Brain	1.6 [0.3;7.0]	1.7 [0.3;6.8]	0.0 [-0.2;0.6]	0.004*	1.7 [0.0;7.4]	1.8 [0.0;7.3]	0.0 [-0.5;2.6]	0.46
Brainstem	4.3 [1.2;27.0]	5.5 [1.1;27.0]	0.3 [-6.4;3.6]	0.001*	2.4 [0.0;29.6]	3.2 [0.0;24.7]	0.0 [-4.9;3.0]	0.96

This table depicts the mean doses (in Gy) in the ST-RT and SCS-RT plans and the differences (i.e., mean dose in ST-RT plan minus mean dose in SCS-RT plan), with both photon and proton therapy. The ST-RT plans were the clinical plans. *Abbreviations:* CL = contralateral (i.e., receiving lowest dose); Gy = Gray; IL = ipsilateral (i.e., receiving highest dose); non-SCR = remaining parotid gland tissue (i.e., parotid gland minus SCR region); PCM = pharyngeal constrictor muscle; RT = radiotherapy; SCR = stem cell rich; SCS = stem cell sparing; ST = standard. * Significant according to Wilcoxon signed-rank test (significance level of p<0.05)..

Table S7. Estimated impact of dose shifts on toxicities (step 2 and 4): comparison of NTCPs (as %) with photons and protons.

	PHOTONS (n=30)				PROTONS (n=15)			
	ST-RT median [range]	SCS-RT median [range]	NTCP difference median [range]	P value	ST-RT median [range]	SCS-RT median [range]	NTCP difference median [range]	P value
**NTCP models by van Rijn-Dekker et al.**(4)								
Patient-reported daytime xerostomia	31.0 [19.4;44.7]	28.9 [17.1;46.8]	-0.9 [-3.8;0.5]	<0.001*	24.9 [14.9;37.3]	25.0 [15.0;36.8]	0.1 [-1.0;1.6]	0.39
Patient-reported eating-related xerostomia	20.7 [10.2;39.4]	19.1 [9.2;32.0]	-1.2 [-7.4;-0.1]	<0.001*	16.7 [7.6;24.8]	16.2 [7.9;24.8]	-0.1 [-1.5;1.1]	0.46
Physician-reported grade ≥2 xerostomia	22.8 [6.6;48.1]	22.0 [6.4;46.1]	-1.8 [-10.7;0.6]	<0.001*	15.8 [7.1;36.2]	15.5 [6.6;35.8]	-0.4 [-1.9;0.9]	0.17
**NTCP models by Van den Bosch et al.**(5)								
Physician-reported grade ≥2 dysphagia	18.7 [3.4;73.2]	17.0 [3.0;71.0]	-0.2 [-4.6;2.5]	0.09	7.5 [2.9;52.9]	7.7 [3.1;55.5]	0.2 [-0.6;2.5]	0.11
Patient-reported aspiration	7.6 [4.0;26.6]	7.6 [3.7;26.3]	-0.1 [-1.4;0.5]	0.08	6.4 [2.3;13.8]	6.4 [2.4;13.9]	0.0 [-0.6;0.9]	0.33
Patient-reported general xerostomia	42.3 [26.2;75.0]	40.5 [24.6;75.3]	-2.1 [-6.1;0.3]	<0.001*	35.1 [19.1;55.6]	35.0 [19.2;56.3]	-0.1 [-2.0;3.3]	0.33
Patient-reported sticky saliva	28.6 [19.0;51.5]	27.5 [17.6;51.2]	-1.1 [-2.7;0.3]	<0.001*	23.1 [17.1;49.2]	23.2 [16.9;48.7]	0.0 [-1.0;1.4]	0.43
Patient-reported taste loss	23.0 [14.2;33.2]	22.1 [13.3;32.9]	-1.0 [-3.3;1.4]	<0.001*	19.0 [8.7;28.2]	18.8 [9.0;28.5]	-0.2 [-0.9;0.4]	0.14
Patient-reported speech problems	18.5 [10.9;51.5]	17.7 [10.9;49.7]	-0.4 [-2.0;1.0]	0.004*	13.5 [7.9;42.1]	13.5 [8.0;42.1]	0.0 [-0.9;0.6]	0.73
Patient-reported oral pain	10.6 [5.8;45.8]	11.1 [5.7;45.2]	0.0 [-1.3;1.3]	0.50	7.9 [5.4;17.1]	8.0 [5.3;17.1]	0.0 [-0.1;0.5]	0.05
Patient-reported nausea and vomiting	11.3 [7.1;17.5]	11.1 [7.0;17.8]	-0.1 [-0.6;0.3]	0.26	6.7 [5.9;9.9]	6.8 [5.9;9.8]	0.0 [-0.1;0.2]	0.40
Patient-reported fatigue	30.7 [15.4;79.1]	30.6 [15.3;79.2]	-0.1 [-0.6;0.4]	0.29	24.4 [13.2;74.6]	24.4 [13.2;74.6]	0.0 [-0.5;0.6]	0.47

This table depicts the NTCPs (as %) in the ST-RT and SCS-RT plans and the differences (i.e., NTCP in ST-RT plan minus NTCP in SCS-RT plan), with both photon and proton therapy. *Abbreviations:* NTCP = normal tissue complication probability; RT = radiation therapy; SCS = stem cell sparing; ST = standard. * Significant according to Wilcoxon signed-rank test (significance level of p<0.05).

Table S8. Consequences for overall sparing of normal tissues (step 2 and 4).

	PHOTONS (n=30)						PROTONS (n=15)					
	**D_{mean} to the body (in Gy)**			**V95 of the body (as %)**			**D_{mean} to the body (in Gy)**			**V95 of the body (as %)**		
**Patient number**	ST-RT	SCS-RT	Δ	ST-RT	SCS-RT	Δ	ST-RT	SCS-RT	Δ	ST-RT	SCS-RT	Δ
**1**	11.73	11.87	0.14	1.32	1.32	0.00	8.96	9.16	0.20	1.84	1.83	-0.01
**2**	12.92	12.43	-0.49	0.81	0.77	-0.04	10.55	11.02	0.47	1.28	1.28	0.00
**3**	8.78	8.90	0.12	0.52	0.55	0.03	4.79	4.77	-0.02	0.45	0.46	0.01
**4**	10.12	9.98	-0.14	0.73	0.79	0.06	8.24	8.24	0.00	1.15	1.15	0.00
**5**	12.17	12.02	-0.15	1.73	1.81	0.08	11.32	11.78	0.46	2.79	2.70	-0.09
**6**	14.82	14.60	-0.22	2.87	2.82	-0.05	11.77	11.81	0.04	3.70	3.61	-0.09
**7**	6.79	6.68	-0.11	0.34	0.36	0.02	5.24	5.24	0.00	0.52	0.52	0.00
**8**	14.73	14.82	0.09	3.86	3.81	-0.05	8.54	8.76	0.22	3.34	3.34	0.00
**9**	13.34	13.67	0.33	0.60	0.95	0.35	5.39	5.34	-0.05	0.94	0.94	0.00
**10**	10.63	10.53	-0.10	0.80	0.72	-0.08	8.18	8.27	0.09	1.11	1.09	-0.02
**11**	11.24	11.07	-0.17	1.16	1.15	-0.01	7.62	7.72	0.10	1.39	1.37	-0.02
**12**	12.46	11.96	-0.50	1.54	1.47	-0.07	12.25	12.18	-0.07	2.48	2.41	-0.07
**13**	8.36	8.51	0.15	0.43	0.45	0.02	4.21	4.21	0.00	0.40	0.39	-0.01
**14**	13.10	13.14	0.04	0.72	0.75	0.03	7.99	7.82	-0.17	0.80	0.81	0.01
**15**	11.12	11.02	-0.10	1.33	1.35	0.02	11.69	11.44	-0.25	2.37	2.46	-0.01
**16**	10.66	10.81	0.15	1.46	1.48	0.02	Not applicable					
**17**	7.99	8.03	0.04	0.62	0.61	-0.01	Not applicable					
**18**	15.60	15.39	-0.21	2.02	2.01	-0.01	Not applicable					
**19**	12.58	12.43	-0.15	1.77	1.85	0.08	Not applicable					
**20**	10.82	10.94	0.12	1.60	1.64	0.04	Not applicable					
**21**	7.48	7.34	-0.14	0.25	0.24	-0.01	Not applicable					
**22**	12.17	12.10	-0.07	0.65	0.66	0.01	Not applicable					
**23**	11.43	11.50	0.07	1.85	1.92	0.07	Not applicable					
**24**	14.07	14.04	-0.03	4.35	4.53	0.18	Not applicable					
**25**	13.62	13.71	0.09	1.61	1.58	-0.03	Not applicable					
**26**	9.48	9.65	0.17	1.16	1.17	0.01	Not applicable					
**27**	9.15	8.88	-0.27	0.46	0.48	0.02	Not applicable					
**28**	5.66	5.82	0.16	0.37	0.35	-0.02	Not applicable					
**29**	13.11	12.67	-0.44	1.95	1.96	0.01	Not applicable					
**30**	6.85	6.77	-0.08	0.15	0.16	0.01	Not applicable					

Abbreviations: Δ = delta (i.e., ST-RT plan minus SCS-RT plan); D_{mean} = mean dose; Gy = Gray; RT = radiotherapy; SCS = stem cell sparing; ST = standard; V95 = percentage of volume receiving 95% of prescribed dose (95% of 70 Gy = 66.50 Gy).

Table S9. Generalizability of SCS-RT (step 3): comparison of mean dose (in Gy) between SCS-RT and ST-RT.

	**Center 1**	**Center 2**	**Center 3**	**Center 4**	**Center 5**	**Center 6**	**Center 7**	**Center 8**	**P value**
	median [range]	median [range]	median [range]	median [range]	median [range]	median [range]	median [range]	median [range]	
**Parotid gland**									
IL SCR region	-2.0 [-3.7;-1.0]	-6.4 [-10.8;-1.7]	-7.2 [-12.6;-2.0]	-2.7 [-10.9;-1.4]	-1.9 [-1.9;-1.8]	-1.6 [-3.0;-0.6]	-1.7 [-2.0;-1.7]	-2.1 [-3.6;-0.4]	0.36
CL SCR region	-4.0 [-4.5;-2.3]	-8.6 [-9.6;-5.2]	-8.4 [-10.1;-6.1]	-4.4 [-7.8;-2.3]	-3.7 [-6.1;-3.2]	0.4 [-2.4;0.7]	-1.7 [-1.9;-0.8]	-2.8 [-3.0;-1.5]	0.01*
IL parotid gland	-1.4 [-3.0;-0.4]	-3.4 [-5.5;-0.3]	-4.0 [-4.9;0.4]	-1.9 [-5.4;-0.7]	-0.8 [-1.6;0.4]	-0.9 [-3.2;0.5]	-0.2 [-0.4;0.2]	0.4 [0.1;1.1]	0.20
CL parotid gland	-2.2 [-3.3;-1.6]	-4.3 [-5.3;-2.7]	-4.0 [-5.6;-3.1]	-2.6 [-4.4;-1.0]	-1.6 [-5.1;-1.2]	0.5 [-2.0;1.2]	-0.5 [-0.8;-0.1]	-0.2 [-0.7;0.2]	0.03*
IL non-SCR region	-1.2 [-2.9;-0.2]	-2.7 [-4.2;0.0]	-2.5 [-4.4;1.1]	-1.7 [-3.9;-0.5]	-0.3 [-1.6;1.0]	-0.4 [-3.7;0.8]	0.2 [0.0;0.8]	1.4 [-0.7;0.2]	0.10
CL non-SCR region	-1.7 [-3.0;-1.3]	-3.2 [-4.0;-2.0]	-2.3 [-4.8;-1.8]	-2.1 [-3.4;-0.7]	-1.0 [-4.8;-0.7]	0.5 [-1.9;1.4]	-0.1 [-0.8;0.4]	0.2 [-0.1;0.7]	0.07
**Other organs at risk**									
IL submandibular gland	0.1 [0.0;0.4]	0.1 [0.0;0.1]	0.2 [-1.1;3.3]	0.1 [-0.7;0.7]	0.0 [-1.0;1.2]	0.5 [-0.6;2.0]	0.1 [-0.3;0.2]	0.5 [0.2;0.8]	0.84
CL submandibular gland	0.5 [0.0;0.5]	-0.3 [-0.4;-0.2]	1.0 [0.2;3.0]	0.3 [-0.1;1.2]	0.4 [-0.9;0.9]	0.7 [-0.8;0.7]	-0.1 [-0.1;0.5]	0.5 [0.0;0.8]	0.27
Oral cavity	0.5 [-0.4;0.7]	0.2 [0.1;1.4]	2.3 [1.4;2.8]	1.5 [-0.5;1.6]	0.8 [0.4;1.3]	0.3 [-0.5;3.7]	0.1 [0.0;0.5]	0.7 [0.2;0.8]	0.22
Superior PCM	-0.1 [-0.5;0.1]	-0.2 [-0.7;0.0]	0.2 [0.1;5.2]	0.1 [-0.1;0.4]	-0.3 [-1.0;-0.1]	0.6 [-0.4;0.7]	0.0 [-0.1;0.3]	0.2 [-0.2;0.4]	0.20
Middle PCM	0.2 [-0.8;1.2]	-0.2 [-2.0;-0.2]	0.5 [-4.4;9.7]	0.8 [0.3;1.1]	-0.1 [-0.2;0.2]	0.8 [0.5;2.4]	0.2 [-0.7;0.5]	0.4 [0.1;1.3]	0.25
Inferior PCM	0.8 [-0.1;1.6]	-0.1 [-0.1;1.2]	0.7 [0.3;2.3]	0.7 [-1.9;0.7]	-0.6 [-3.4;1.6]	2.6 [0.9;3.8]	0.2 [0.1;0.3]	0.2 [0.0;0.6]	0.20
Supraglottic area	0.7 [0.1;1.0]	0.0 [-0.3;0.4]	2.4 [-9.1;3.7]	0.6 [-1.4;1.4]	1.6 [1.2;3.1]	3.6 [-0.1;5.7]	0.3 [0.0;0.6]	0.1 [-0.2;0.3]	0.43
Brain	0.0 [0.0;0.0]	0.0 [-0.2;0.0]	0.1 [0.0;0.3]	0.0 [0.0;0.1]	0.0 [-0.1;0.0]	0.0 [0.0;0.2]	0.0 [0.0;0.1]	0.0 [-0.3;0.0]	0.51
Brainstem	-0.1 [-0.2;-0.1]	0.0 [-0.5;0.0]	0.4 [0.1;0.9]	0.1 [0.1;0.2]	0.0 [-0.2;0.1]	0.0 [-0.2;0.2]	0.3 [0.1;0.6]	-0.2 [-1.0;0.1]	0.05*

This table depicts the mean dose differences between SCS-RT and ST-RT with photon therapy (i.e., mean dose in ST-RT plan minus mean dose in SCS-RT plan). The ST-RT plans were optimized according to the center's own standard practice. *Abbreviations:* CL = contralateral (i.e., receiving lowest dose); Gy = Gray; IL = ipsilateral (i.e., receiving highest dose); non-SCR = remaining parotid gland tissue (i.e., parotid gland minus SCR region); PCM = pharyngeal constrictor muscle; RT = radiotherapy; SCR = stem cell rich; SCS = stem cell sparing; ST = standard.

Table S10. Multicenter study (step 3): raw mean dose data (in Gy).

	**Ipsilateral SCR region**		**Contralateral SCR region**	
	ST-RT median [range]	SCS-RT median [range]	ST-RT median [range]	SCS-RT median [range]
Center 1	25.1 [19.5;39.0]	23.1 [15.9;38.0]	17.0 [10.3;17.4]	12.8 [7.9;12.9]
Center 2	30.9 [30.7;38.0]	24.3 [20.1;36.3]	28.4 [19.8;29.5]	19.7 [14.6;19.9]
Center 3	29.2 [26.3;41.9]	22.0 [13.7;39.8]	24.3 [23.1;24.5]	15.9 [14.4;17.0]
Center 4	26.3 [20.0;36.9]	18.7 [15.4;34.3]	15.0 [13.5;21.9]	12.7 [9.2;14.1]
Center 5	22.6 [17.5;35.5]	20.8 [15.5;33.7]	15.6 [14.5;18.9]	12.4 [8.5;15.3]
Center 6	16.7 [15.9;27.2]	16.0 [14.3;24.3]	9.0 [6.0;15.7]	9.7 [6.4;13.4]
Center 7	19.9 [14.2;34.8]	18.0 [12.5;33.2]	11.7 [7.4;12.3]	10.1 [6.6;10.4]
Center 8	27.6 [16.6;40.7]	24.0 [14.5;40.3]	13.0 [8.6;13.8]	10.2 [7.1;10.8]
	**Ipsilateral parotid gland**		**Contralateral parotid gland**	
	ST-RT median [range]	SCS-RT median [range]	ST-RT median [range]	SCS-RT median [range]
Center 1	25.7 [23.6;29.9]	22.7 [22.2;29.5]	17.7 [13.5;24.0]	15.4 [11.9;20.7]
Center 2	29.3 [27.7;31.8]	26.3 [24.3;29.0]	26.2 [19.4;29.3]	20.9 [16.8;25.1]
Center 3	28.2 [27.6;33.6]	24.2 [22.7;34.1]	23.9 [23.4;27.8]	20.2 [18.3;23.8]
Center 4	28.3 [20.3;28.8]	22.9 [19.6;26.8]	15.7 [15.1;25.4]	14.7 [12.5;21.0]
Center 5	23.5 [22.9;27.9]	23.4 [22.7;26.3]	20.1 [18.0;22.2]	18.5 [12.9;21.0]
Center 6	21.8 [18.2;22.5]	19.3 [18.7;20.9]	10.9 [8.5;21.0]	12.1 [9.0;19.0]
Center 7	19.5 [19.1;26.3]	19.1 [18.9;26.6]	13.9 [10.7;18.4]	13.4 [9.9;18.3]
Center 8	25.7 [21.9;31.4]	26.1 [22.0;32.5]	14.9 [11.7;19.5]	14.2 [11.8;19.3]
	**Ipsilateral non-SCR region**		**Contralateral nonSCR region**	
	ST-RT median [range]	SCS-RT median [range]	ST-RT median [range]	SCS-RT median [range]
Center 1	27.3 [23.2;27.5]	24.4 [21.9;27.3]	17.8 [14.4;25.7]	16.1 [13.1;22.6]
Center 2	27.1 [27.0;32.1]	27.1 [24.3;27.9]	25.2 [19.3;29.6]	21.2 [17.3;26.4]
Center 3	29.1 [27.2;31.4]	26.7 [22.8;32.5]	23.7 [23.4;28.2]	21.2 [18.9;26.4]
Center 4	26.6 [20.3;28.9]	24.9 [19.8;25.0]	16.0 [15.5;26.4]	15.3 [13.5;22.9]
Center 5	25.0 [23.0;26.0]	24.4 [24.0;24.7]	20.4 [19.0;23.9]	19.4 [14.2;23.1]
Center 6	20.4 [18.7;24.1]	20.0 [19.5;20.5]	11.4 [9.2;22.3]	12.8 [9.7;20.5]
Center 7	20.3 [19.3;24.1]	20.5 [19.3;24.8]	14.5 [11.7;19.9]	14.3 [10.9;20.3]
Center 8	25.4 [24.0;29.0]	26.8 [24.4;30.5]	15.3 [12.6;21.4]	15.2 [13.2;21.6]
	**Ipsilateral submandibular gland**		**Contralateral submandibular gland**	
	ST-RT median [range]	SCS-RT median [range]	ST-RT median [range]	SCS-RT median [range]
Center 1	58.4 [46.3;66.7]	58.5 [46.2;67.1]	37.7 [37.2;42.4]	38.2 [37.7;42.5]
Center 2	60.6 [46.0;66.0]	60.6 [46.2;66.1]	43.1 [42.6;47.4]	42.8 [42.4;47.0]
Center 3	62.1 [49.9;69.3]	61.0 [53.2;69.5]	44.9 [42.7;45.2]	46.3 [42.9;47.8]
Center 4	54.9 [42.6;66.6]	55.0 [41.9;67.3]	35.4 [31.4;39.9]	36.7 [31.7;39.8]
Center 5	56.3 [40.6;66.9]	55.3 [41.8;66.8]	37.6 [13.6;38.1]	38.5 [12.7;38.5]
Center 6	52.6 [37.5;69.7]	52.0 [39.5;70.2]	33.5 [32.5;33.6]	34.2 [31.7;34.2]
Center 7	57.4 [44.0;66.1]	57.6 [44.1;65.8]	34.4 [31.0;36.4]	34.3 [30.9;36.9]
Center 8	63.8 [43.0;67.7]	64.0 [43.9;68.3]	39.3 [39.2;41.2]	40.0 [39.3;41.7]

	**Oral cavity**		**Superior PCM**	
	ST-RT median [range]	SCS-RT median [range]	ST-RT median [range]	SCS-RT median [range]
Center 1	40.8 [35.7;41.6]	41.2 [36.3;41.5]	62.7 [58.2;65.0]	62.6 [58.4;64.6]
Center 2	43.9 [41.4;47.2]	44.0 [42.8;47.5]	64.4 [64.0;64.6]	64.0 [63.9;64.2]
Center 3	41.9 [37.4;44.8]	44.8 [39.7;46.3]	62.3 [60.2;67.7]	65.4 [62.6;67.9]
Center 4	33.7 [31.9;39.6]	35.2 [33.5;39.1]	61.7 [57.7;63.2]	61.6 [58.0;63.3]
Center 5	39.2 [34.6;42.8]	39.6 [35.9;43.6]	63.4 [62.4;64.8]	63.1 [61.3;64.7]
Center 6	43.3 [31.0;43.5]	42.8 [34.7;43.7]	63.9 [62.6;64.2]	63.7 [63.1;64.6]
Center 7	33.0 [30.6;34.7]	33.2 [31.1;34.6]	61.7 [60.8;62.8]	61.7 [60.7;63.2]
Center 8	47.7 [43.2;48.0]	48.2 [43.9;48.5]	64.0 [63.0;65.4]	63.8 [63.4;65.5]
	**Middle PCM**		**Inferior PCM**	
	ST-RT median [range]	SCS-RT median [range]	ST-RT median [range]	SCS-RT median [range]
Center 1	40.6 [39.9;47.9]	40.8 [39.1;49.1]	25.5 [24.9;28.8]	25.7 [25.4;30.3]
Center 2	54.1 [49.6;57.0]	53.9 [47.6;56.7]	47.9 [47.8;49.1]	49.0 [47.9;49.0]
Center 3	44.9 [44.7;48.6]	45.2 [44.2;54.6]	38.0 [35.7;38.9]	38.7 [36.0;41.1]
Center 4	36.1 [31.6;47.3]	36.3 [32.4;48.4]	25.7 [22.1;29.9]	23.8 [22.7;30.6]
Center 5	48.2 [47.3;53.6]	48.4 [47.1;53.4]	38.3 [32.2;41.2]	34.9 [31.6;42.8]
Center 6	46.0 [45.8;52.4]	48.2 [46.5;53.2]	34.8 [31.8;36.6]	35.7 [34.5;40.4]
Center 7	36.5 [34.3;43.9]	37.0 [34.5;43.2]	19.3 [18.5;23.6]	19.4 [18.7;23.8]
Center 8	42.6 [42.5;46.4]	43.8 [42.6;46.8]	33.8 [32.4;35.8]	33.8 [33.0;36.0]
	**Supraglottic area**		**Brain**	
	ST-RT median [range]	SCS-RT median [range]	ST-RT median [range]	SCS-RT median [range]
Center 1	26.9 [16.7;29.3]	27.6 [16.8;30.3]	0.9 [0.7;2.7]	0.9 [0.7;2.6]
Center 2	42.6 [40.0;43.7]	42.6 [40.4;43.3]	1.1 [0.8;3.0]	1.1 [0.8;2.8]
Center 3	33.7 [24.7;35.2]	27.1 [26.0;37.3]	0.6 [0.3;1.8]	0.7 [0.4;2.0]
Center 4	24.1 [15.2;30.9]	25.5 [13.8;31.5]	0.9 [0.7;2.0]	0.9 [0.7;2.1]
Center 5	41.8 [20.3;43.2]	43.0 [23.4;44.8]	2.5 [1.2;9.5]	2.5 [1.2;9.5]
Center 6	37.9 [18.6;40.5]	40.4 [24.3 [41.5]	1.3 [1.0;2.7]	1.2 [1.0;2.9]
Center 7	30.5 [14.9;23.3]	30.7 [15.5;34.5]	0.8 [0.6;2.2]	0.8 [0.6;2.3]
Center 8	33.6 [27.7;36.5]	33.7 [27.6;36.9]	0.7 [0.7;2.3]	0.8 [0.7;2.0]

	**Brainstem**	
	ST-RT median [range]	SCS-RT median [range]
Center 1	3.8 [2.6;8.5]	3.7 [2.5;8.3]
Center 2	3.2 [2.3;9.3]	3.2 [2.3;8.8]
Center 3	3.1 [1.8;5.3]	3.5 [1.8;6.3]
Center 4	2.6 [1.8;4.4]	2.7 [1.9;4.6]
Center 5	3.1 [2.4;5.1]	2.9 [2.4;5.3]
Center 6	2.8 [2.1;5.5]	2.7 [2.1;5.7]
Center 7	3.4 [2.2;6.5]	3.9 [2.3;6.9]
Center 8	2.2 [2.2;6.5]	2.2 [2.1;5.5]

Abbreviations: Gy = Gray; non-SCR = remaining parotid gland tissue (i.e., parotid gland minus SCR region); OAR = organ at risk; PCM = pharyngeal constrictor muscle; RT = radiotherapy; SCS = stem cell sparing; SCR = stem cell rich; ST = standard.

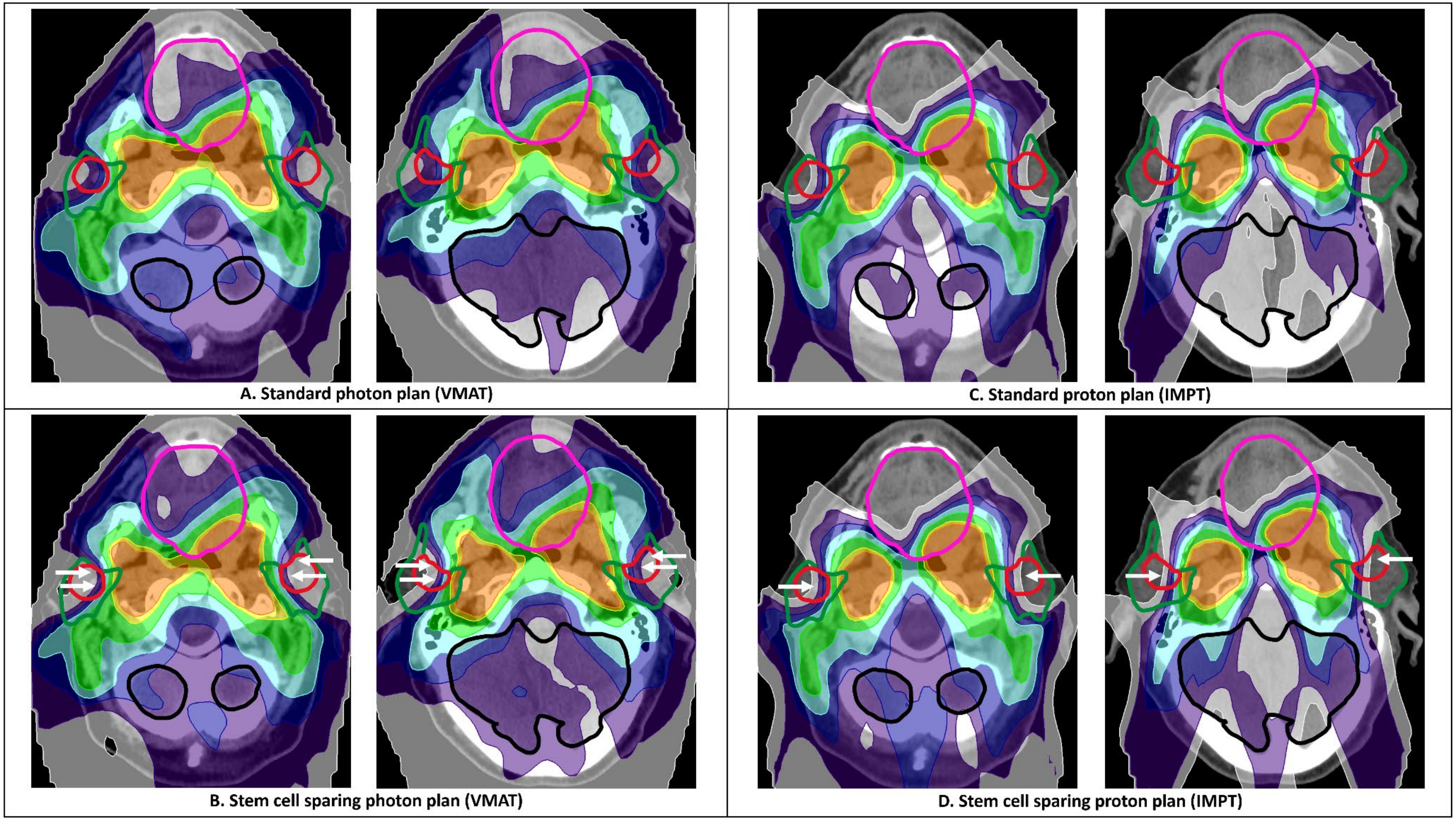

Figure S1. Differences in dose distributions. An example dose distribution with different techniques (orange = 66.5 Gy; yellow = 60 Gy; green = 51.54 Gy; light blue = 40 Gy; dark blue = 30 Gy; purple = 20 Gy; white = 10 Gy). The following OARs were delineated: stem cell rich regions (red), parotid glands (green), oral cavity (pink), and brain (black). *Abbreviations:* Gy = Gray; IMPT = intensity modulated proton therapy; VMAT = volumetric modulated arc therapy.

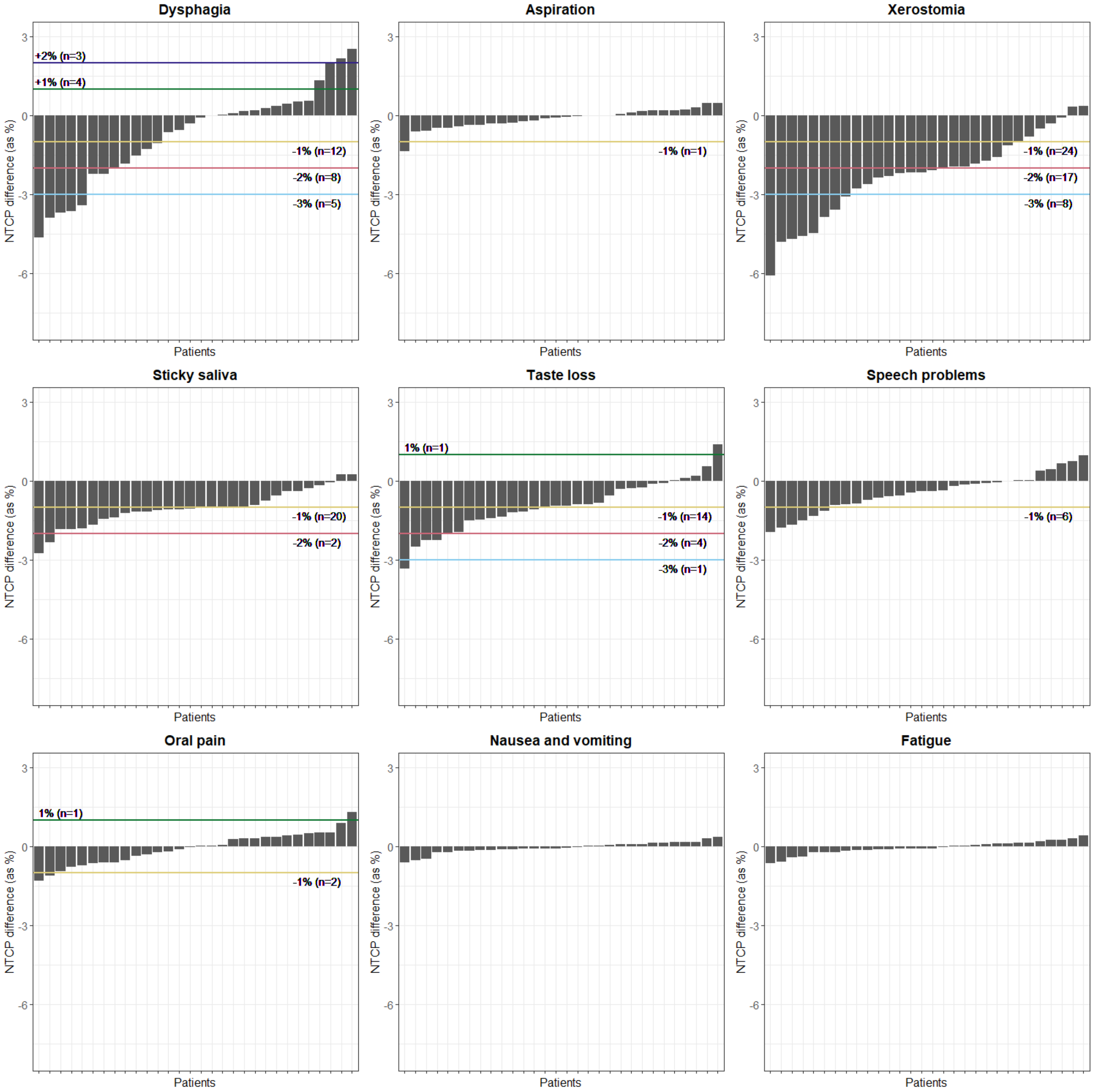

Figure S2. Estimated clinical impact of SCS-RT on several side-effects. This figure depicts the NTCP differences (i.e., NTCP in ST-RT plan minus NTCP in SCS-RT plan) for several xerostomia outcomes. The NTCPs were calculated using the models developed by Van den Bosch et al.[5] The lines depict the number of patients in which the NTCP changed with at least +2% (purple), +1% (green), -1% (yellow), -2% (red) and -3% (blue). Abbreviations: NTCP = normal tissue complication probability; SCS = stem cell sparing; ST = standard; RT = radiotherapy.

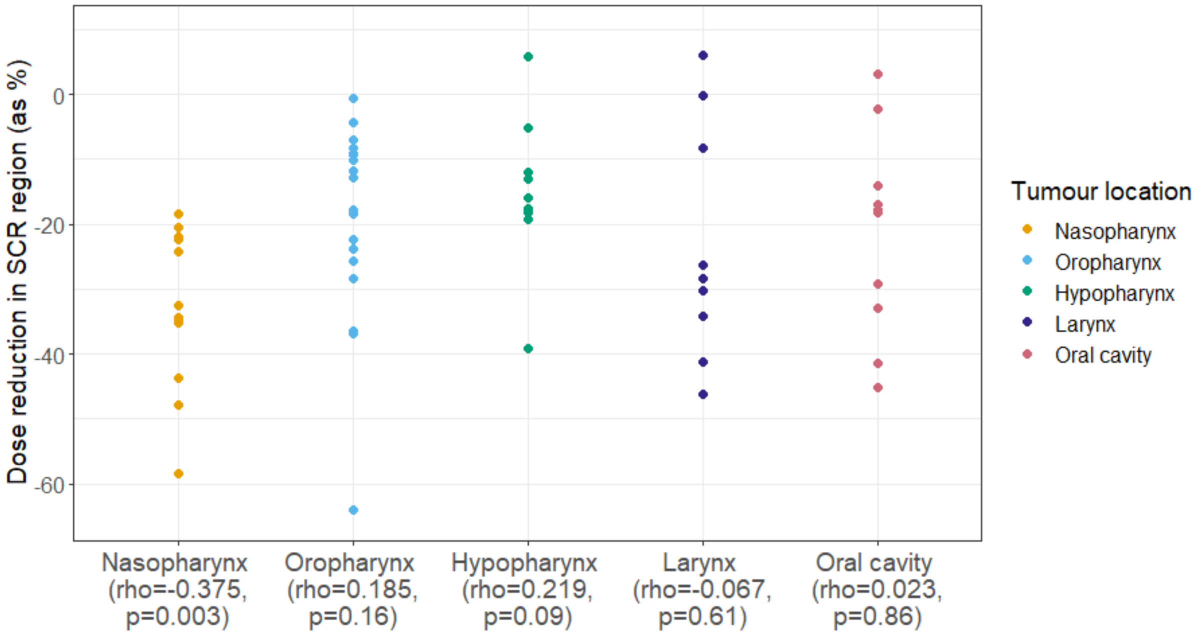

Figure S3. Achieved dose reduction in SCR regions: tumor location. This figure depicts the achieved dose reductions in the SCR regions (as percentage) per tumor location. For this analysis, the ipsilateral and contralateral SCR regions were analyzed combined. *Abbreviations:* rho = Spearman's rank correlation coefficient; SCR = stem cell rich.

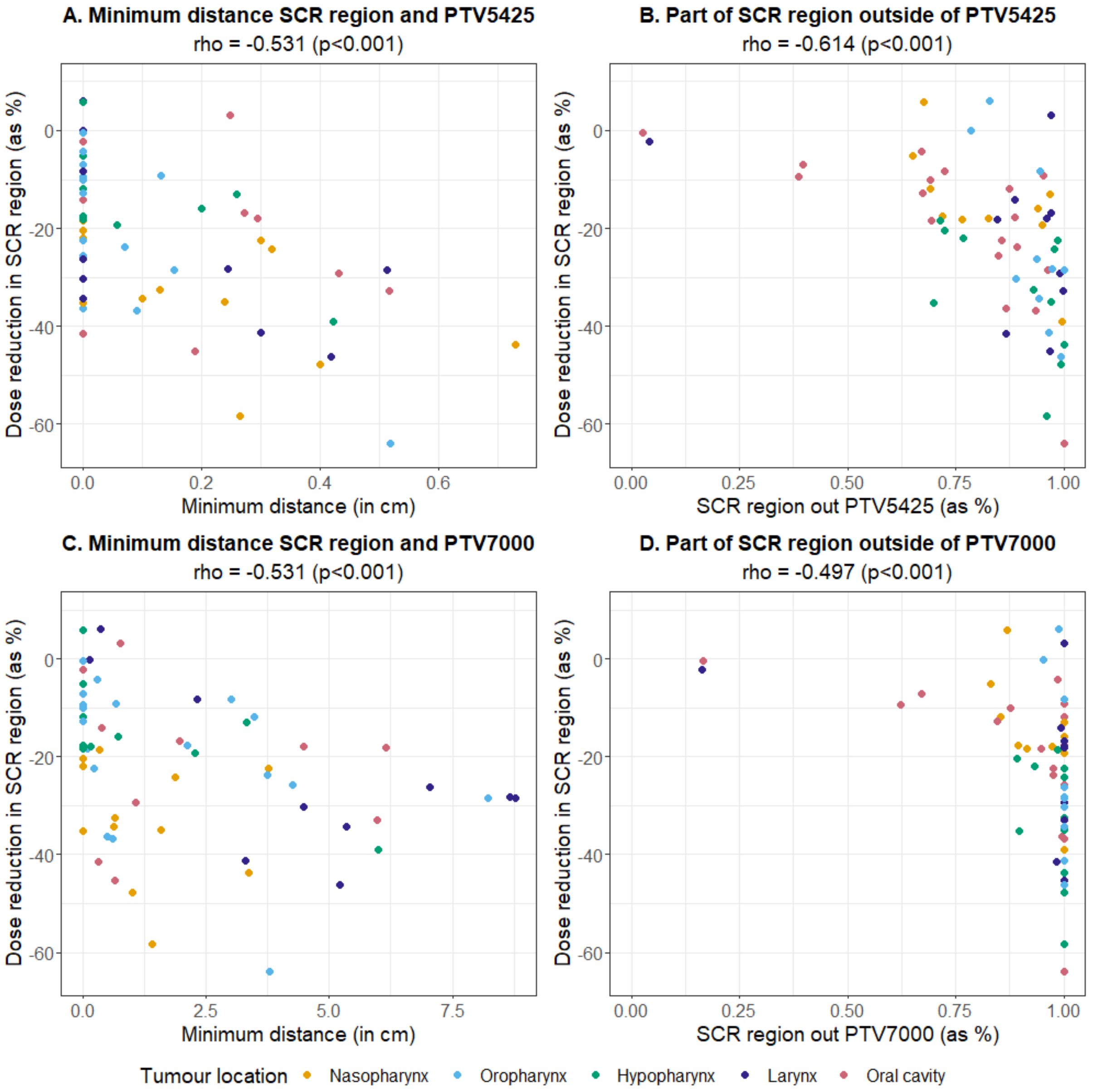

Figure S4. Achieved dose reduction in SCR regions: distance to target volume. This figure depicts the achieved dose reductions in the SCR regions (as percentage) per minimum distance to target volume (A and C) and per part of SCR region outside target volume (as percentage using the method as proposed by Tambas et al.[6], B and C). For this analysis, the ipsilateral and contralateral SCR regions were analyzed combined. *Abbreviations:* PTV5425 = planning target volume to 54.25 Gy (i.e., elective volume); PTV7000 = planning target volume to 70.00 Gy (i.e., tumor volume); rho = Spearman's rank correlation coefficient; SCR = stem cell rich.

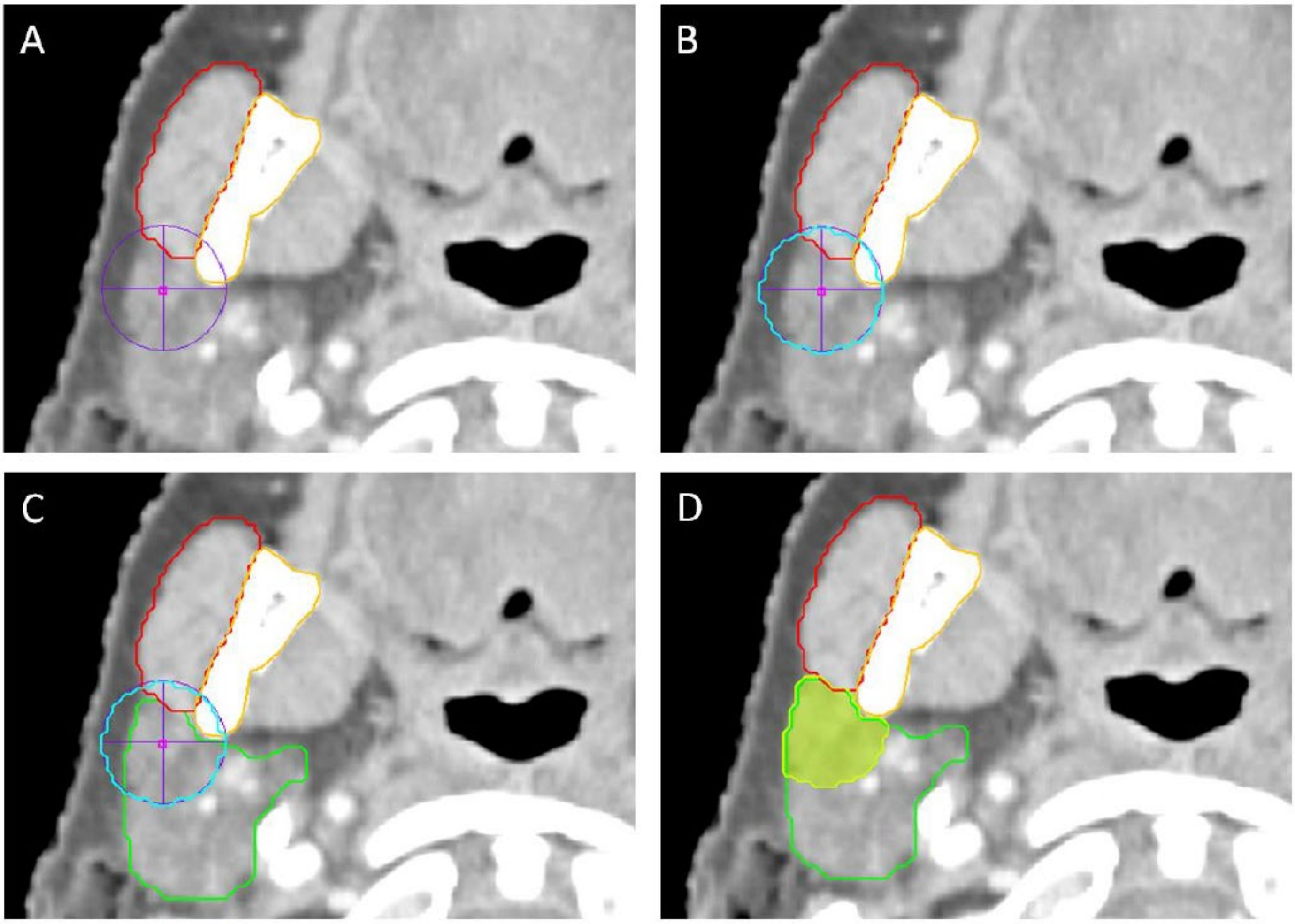

Figure S5. Delineation of the stem cell rich region. Transversal computed tomography images with masseter muscle (red line) and mandible (orange line). (A) Automatically generated point with probably highest concentration stem cells (red dot) based on the publication of "Anonymized for Review" (1). (B) Margin around generated stem cell point (blue line). (C) Margin around generated stem cell point and parotid gland (green line). (D) Stem cell rich (SCR) region (light green area).

References

1. van Rijn – Dekker MI, la Bastide – van Gemert S, Stokman MA, Vissink A, Coppes RP, Langendijk JA, et al. Radiation-induced xerostomia is related to stem cell dose-dependent reduction of saliva production. International Journal of Radiation Oncology*Biology*Physics [Internet]. 2024 Apr; Available from: https://linkinghub.elsevier.com/retrieve/pii/S036030162400511X
2. Brouwer CL, Steenbakkers RJHM, Bourhis J, Budach W, Grau C, Grégoire V, et al. CT-based delineation of organs at risk in the head and neck region: DAHANCA, EORTC, GORTEC, HKNPCSG, NCIC CTG, NCRI, NRG Oncology and TROG consensus guidelines. Radiotherapy and Oncology [Internet]. 2015 Oct;117(1):83–90. Available from: https://linkinghub.elsevier.com/retrieve/pii/S0167814015004016
3. Steenbakkers RJHM, van Rijn–Dekker MI, Stokman MA, Kierkels RGJ, van der Schaaf A, van den Hoek JGM, et al. Parotid Gland Stem Cell Sparing Radiation Therapy for Patients With Head and Neck Cancer: A Double-Blind Randomized Controlled Trial. International Journal of Radiation Oncology*Biology*Physics [Internet]. 2022 Feb;112(2):306–16. Available from: https://pubmed.ncbi.nlm.nih.gov/34563635/
4. van Rijn–Dekker MI, van Luijk P, Schuit E, van der Schaaf A, Langendijk JA, Steenbakkers RJHM. Prediction of Radiation-Induced Parotid Gland–Related Xerostomia in Patients With Head and Neck Cancer: Regeneration-Weighted Dose. International Journal of Radiation Oncology*Biology*Physics [Internet]. 2023 May; Available from: https://linkinghub.elsevier.com/retrieve/pii/S0360301623004376
5. Van den Bosch L, van der Schaaf A, van der Laan HP, Hoebers FJP, Wijers OB, van den Hoek JGM, et al. Comprehensive toxicity risk profiling in radiation therapy for head and neck cancer: A new concept for individually optimised treatment. Radiotherapy and Oncology [Internet]. 2021 Apr;157:147–54. Available from: https://linkinghub.elsevier.com/retrieve/pii/S0167814021060217
6. Tambas M, van der Laan HP, Rutgers W, van den Hoek JGM, Oldehinkel E, Meijer TWH, et al. Development of advanced preselection tools to reduce redundant plan comparisons in model-based selection of head and neck cancer patients for proton therapy. Radiotherapy and Oncology [Internet]. 2021 Jul;160:61–8. Available from: https://linkinghub.elsevier.com/retrieve/pii/S0167814021061946
